# Treatment of Typhoid Fever

**Published:** 1904-10

**Authors:** Robert M. Sterrett

**Affiliations:** New York, N. Y., Former Demonstrator of Surgical Pathology, College Physicians and Surgeons, Chicago; Former Attending Physician, West Side Dispensary, Medical Department, Chicago


For Texas Medical Journal.

### Treatment of Typhoid Fever.

BY ROBERT M. STERRETT, M. D., NEW YORK, N. Y.,
Former Demonstrator of Surgical Pathology, College Physicians and Surgeons, Chicago; Former Attending Physician, West Side Dispensary, Medical Department, Chicago.

To speak of the treatment of so complicated a disease as that represented by typhoid fever, in any concise, specific way, is scarcely proper; and to present a definite, comprehensive line of therapy in a few words is not possible.

At the same time, as our knowledge of the etiology and pathology of disease is perfected, our comprehension and scientific application of remedial measures is more definite and precise, we can successfully cope with heretofore exceedingly difficult problems in medicine to the great saving of life and to the honor and satisfaction of the physician.

In mapping out a line of treatment for typhoid fever, with all its symptoms; its stages of onset, progress and decline; its complications and sequelæ, it is of the greatest importance that we comprehend early in the campaign the causes, locale, and mode of development of the infection.

That this disease is always associated with the propagation of the typhoid bacillus, and that the small intestine is the place where the bacillary development occurs, is agreed by all physicians worthy the name.

That there are other factors in the full development of typhoid fever than the bacilli is shown from the fact that under certain conditions unfavorable to the bacilli, but unfavorable to the host, the former are disposed of by the natural physiological processes resident in the normal healthy individual, and the disease does not become manifest.

If, on the other hand, the invasion of the microbic army is of such force and numbers, and the condition of the host such that the natural powers are enfeebled, the disease processes are set up which, unless interfered with, pursue the usual cycles of typhoid fever, of greater or less severity.

It is the province of the physician to "interfere" with these disease processes whenever it is possible, otherwise a good nurse is all a patient suffering with typhoid or any other disease requires to go through the ordeal—and, unfortunately, this is what some patients get, plus the regular visits of the doctor who "looks in" every day to see how the sick man is progressing.

The way some of our dogmas do possess us is amazing. When we once get the idea that a person infected with a colony of bacteria *must* pass through all the stages of disease resulting from the birth, growth and death of any number of these bacteria, we don't care, some of us, to interfere, but stand by and note the *laws established* by the enemy we are sworn to conquer, as they affect the charges we have undertaken to defend. And, too, because we would rather *wait* until we see the enemy in our back yard before we decide whether he is really a foe or a friend; in other words, until we can be sure of our diagnosis—until the thing has gone so far we are sure of it. Then, if the patient dies, we *know* he died of typhoid fever; if he get well, we are *sure* he got well of typhoid fever, and we're very glad and proud of what? Our diagnosis! How about doing something to reduce the force and numbers of bacilli in the very beginning—when we, from experience and observation, realize the chances are that the invasion is in its incipiency, and there is yet an opportunity to do something to check it?

Here is where we can sum up—concentrate. We know that the principal lesions are in the intestinal tract; that the bacilli swarm there; that they increase the power of putrefactive bacilli there also, and which in turn reciprocate; that, if we can even render the intestines less *fit* for bacillary propagation, we can do some good, and, striking at the "main issue of the campaign," we can reduce the total of damage likely to occur, and prevent complications and sequelæ.

Intestinal antisepsis is the keynote to the situation. The sulphocarbolates are, in my opinion and practice, the safest, most efficient and reliable, when used boldly and in sufficient dosage to render the stools inodorous—no matter what amount may be required in any given case.

If there is little diarrhea, the sulphocarbolates of sodium are indicated; if the discharges are excessive, the zinc salt is used; if there has been much emaciation or a cachexia, the sulphocarbolate of lime is of great service. That these salts do render the bowels decidedly antiseptic is without a doubt, as evidenced by the reduction of temperature; the changes in the character and the frequency of the dejecta; and the general shortening of the time of the disease, when they are used early in the attack.

The various symptoms, headache, delirium, etc., are greatly relieved when the intestinal tract is cleaned out and kept clean by the administration of calomel and mild salines early in the treatment, and these followed by the free use of the sulphocarbolates.

The dose is anywhere from five to ten grains of either of the salts, or a tablet of the three combined—five grains daily until the desired results are obtained. In children the dose may be from one to three grains and given every two to four hours, until the bowels become aseptic, the tympanites disappear, and there is general improvement manifest.

This line of treatment brings good results; all theories of the impracticability of rendering the prima via aseptic to the contrary notwithstanding. It is safe—if the sulphocarbolates are pure—and to any members of the cult who would prefer to see their typhoid fever cases get well, even if they do not show all the classic evidences of the disease as laid down in the books, I would say: unless you know some better way, try this. If you do, let us have it, by all means.

